# Comparison of the effects of intravenous, enteral and enteral + intravenous supply of glutamine on malnutrition in sepsis

**DOI:** 10.1186/cc9805

**Published:** 2011-03-11

**Authors:** G Koksal, G Karaören, H Akarcay, E Karabulut, Y Tunali, S Vehid, H Oz

**Affiliations:** 1 I.U Cerrahpasa Medical Faculty, Istanbul, Turkey

## Introduction

Our aim was to compare the effects of intravenous, enteral and intravenous + enteral supplemented glutamine on prediction of positive feeding parameters (transferrin, nitrogen balance and creatine/height index) for malnutrition in septic patients.

## Methods

This was a prospective, randomized, controlled, single-blind, clinical study. Forty septic patients with malnutrition were randomly divided into four groups (*n *= 10 each group). All patients were receiving enteral access, and had a clinical diagnosis of either severe sepsis or septic shock. All patients received enteral nutrition during 15 days. Enteral feeding was delivered at a constant rate to achieve energy expenditure (Harris-Benedict equation). Blood and urine samples were obtained for transferrin, nitrogen balance and creatine/height index at least at baseline and on study days 7 and 15. Group 1: received 30 g/day intravenous glutamine, Group 2: received 30 g/day enteral glutamine, Group 3: received 15 g/day enteral + 15 g/day intravenous glutamine, Group 4: control group, without glutamine only enteral feeding. Data were compared by the Tukey HSD test.

## Results

Nitrogen balance levels were not significantly different between groups on the first 7 and 15 days. The transferrin level was higher in Group 2 than Group 4 on the first 7 days (*P *< 0.001). Transferrin levels were not significantly different between the other groups. Transferrin levels were higher in Group 3 than Group 2 (*P *< 0.05) and Group 4 (*P *< 0.001) in 15 days. Creatine/height index was higher in Group 3 than Group 4 (*P *< 0.05) in 15 days.

## Conclusions

Enteral plus intravenous supplemented glutamine has more beneficial effects on transferrin and creatine/height index than only enteral or intravenous supply of glutamine. Also, we observed that enteral feeding of supplemented glutamine has beneficial effects on transferrin, nitrogen balance and creatine/height index in Groups 1, 2 and 3 when compared with Group 4.
